# Global burden of disease due to smokeless tobacco consumption in adults: analysis of data from 113 countries

**DOI:** 10.1186/s12916-015-0424-2

**Published:** 2015-08-17

**Authors:** Kamran Siddiqi, Sarwat Shah, Syed Muslim Abbas, Aishwarya Vidyasagaran, Mohammed Jawad, Omara Dogar, Aziz Sheikh

**Affiliations:** Department of Health Sciences, Hull York Medical School, University of York, Room 105a, First floor, ARRC Building, Heslington, York, YO10 5DD UK; Fatima Memorial Hospital College of Medicine and Dentistry, Fatima Memorial System, Shadman, Lahore, 48000 Pakistan; Department of Primary Care and Public Health, Imperial College London, Charing Cross Campus, Reynold’s Building, Hammersmith, W6 8RP UK; Usher Institute of Population Health Sciences and Informatics, The University of Edinburgh, Medical School Doorway 3, Teviot Place, Edinburgh, EH8 9AG UK

## Abstract

**Background:**

Smokeless tobacco is consumed in most countries in the world. In view of its widespread use and increasing awareness of the associated risks, there is a need for a detailed assessment of its impact on health. We present the first global estimates of the burden of disease due to consumption of smokeless tobacco by adults.

**Methods:**

The burden attributable to smokeless tobacco use in adults was estimated as a proportion of the disability-adjusted life-years (DALYs) lost and deaths reported in the 2010 Global Burden of Disease study. We used the comparative risk assessment method, which evaluates changes in population health that result from modifying a population’s exposure to a risk factor. Population exposure was extrapolated from country-specific prevalence of smokeless tobacco consumption, and changes in population health were estimated using disease-specific risk estimates (relative risks/odds ratios) associated with it. Country-specific prevalence estimates were obtained through systematically searching for all relevant studies. Disease-specific risks were estimated by conducting systematic reviews and meta-analyses based on epidemiological studies.

**Results:**

We found adult smokeless tobacco consumption figures for 115 countries and estimated burden of disease figures for 113 of these countries. Our estimates indicate that in 2010, smokeless tobacco use led to 1.7 million DALYs lost and 62,283 deaths due to cancers of mouth, pharynx and oesophagus and, based on data from the benchmark 52 country INTERHEART study, 4.7 million DALYs lost and 204,309 deaths from ischaemic heart disease. Over 85 % of this burden was in South-East Asia.

**Conclusions:**

Smokeless tobacco results in considerable, potentially preventable, global morbidity and mortality from cancer; estimates in relation to ischaemic heart disease need to be interpreted with more caution, but nonetheless suggest that the likely burden of disease is also substantial. The World Health Organization needs to consider incorporating regulation of smokeless tobacco into its Framework Convention for Tobacco Control.

**Electronic supplementary material:**

The online version of this article (doi:10.1186/s12916-015-0424-2) contains supplementary material, which is available to authorized users.

## Background

Smokeless tobacco (SLT) consists of a number of products containing tobacco, which are consumed—without burning—through the mouth or nose [1]. A diverse range of SLT products are available worldwide, varying in their composition, methods of preparation and consumption, and associated health risks (Table 1) [1]. Its use is most prevalent in South and South-East Asia where one-third of tobacco is consumed in smokeless form [2, 3]. Wrapped in a betel leaf with areca nut, slaked lime, and catechu, SLT is often served at social occasions in this region. Other products (e.g. gutkha, khaini) contain slaked lime, areca nut, flavourings, and aromatic substances [4]. A number of products based on powdered tobacco (e.g. snus) are also consumed in Nordic countries and North America. In other parts of world, the most commonly used SLT products (Table 1) include Chimó (Venezuela), Nass (Uzbekistan, Kyrgyzstan), Tambook (Sudan, Chad), and Snuff (Nigeria, Ghana, South Africa).

**Table 1:** Smokeless tobacco products consumed most commonly across the world

Smokeless tobacco products	Regions (WHO)	Countries (highest consumption)	Other ingredients	Preparation and use	pH^a^	Nicotine^a^ (mg/g)	Total TSNA^a^ (ng/g)
Snus (Swedish)	Europe (Region A)	Nordic countries (Denmark, Finland, Iceland, Norway, Sweden)	Water, sodium carbonate, sodium chloride, moisturisers, flavouring	A heat treatment process; placed between the gum and upper lip	6.6–7.2	7.8–15.2	601–723
Plug, Snuff (US), Snus (US)	Americas (Region A and B)	US, Canada, Mexico	Sweeteners, liquorice	Plug; air cured	4.7–7.8	3.9–40.1	313–76,500
				Dry or moist snuff; finely ground and fire cured			
				Snus; steam cured			
				Snuff; kept between lip and gum, dry snuff can be inhaled too			
Chimó	Americas (Region B)	Venezuela, Colombia	Sodium bicarbonate, brown sugar, Mamo’n tree ashes	Tobacco paste made from tobacco leaves; placed between the lip or cheek and gum and left there for some time	6.9–9.4	5.3–30.1	9390
Nass (Naswar)	Europe (Region B) and Eastern Mediterranean (Region D)	Uzbekistan, Kyrgyzstan, Tajikistan, Afghanistan, Pakistan, Iran	Lime, ash, flavourings (cardamom), indigo	Sundried and powdered; placed between lip or cheek and gum	8.4–9.1	8.9–14.2	478–1380
Tambook	Eastern Mediterranean (Region D) and Africa (Region D)	Sudan, Chad	Mixed with moist sodium bicarbonate	Fermented and grounded; placed and kept in mouth	7.3–10.1	9.6–28.2	302,000–992,000
Snuff (North and West African)	Africa (Region D)	Nigeria, Ghana, Algeria, Cameroon, Chad, Senegal	Dried tobacco leaves mixed with potassium nitrate and other salts	Dry snuff; finely ground and inhaled as a pinch	9.0–9.4	2.5–7.4	1520–2420
				Moist snuff is placed in mouth			
Snuff (South African)	Africa (Region E)	South Africa	Dried tobacco leaves mixed with ash	Dry snuff; finely ground and inhaled as a pinch	6.5–10.1	1.2–17.2	1710–20,500
Khaini	South East Asia (Regions B and D) Western Pacific (Region B) Eastern Mediterranean (Region D) Europe (Region A)	India, Bangladesh, Nepal, Bhutan	Slaked lime, menthol, flavourings, areca nut	Shredded; kept in mouth between lips and gum	9.6–9.8	2.5–4.8	21,600–23,900
Zarda		Bangladesh, India, Pakistan, Myanmar, Thailand, Indonesia, Nepal, Maldives, Sri Lanka, UK	Served wrapped in a betel leaf with lime, catechu, areca nuts	Shredded tobacco leaves are boiled with lime and saffron; the mixture is dried then chewed and spat	5.2–6.5	9.5–30.4	5490–53,700
Gutkha		India, Pakistan, Bangladesh, Nepal, Myanmar, Sri Lanka, UK	Betel nut, catechu, flavourings, sweeteners	Commercially manufactured; sucked, chewed, and spat	7.4–8.9	0.2–4.2	83–23,900

*WHO* World Health Organization, *TSNA* tobacco-specific nitrosamines

^a^Figures are adapted from Stanfill et al. [6], Lawler et al. [17], and NIH & CDC 2014 report on smokeless tobacco products [37]

In addition to nicotine, SLT products contain over 30 carcinogens [5] including tobacco-specific nitrosamines (TSNA), arsenic, beryllium, cadmium, nickel, chromium, nitrite, and nitrate. The level of nicotine and carcinogens vary between products (Table 1) [6]. For example, nicotine content among SLT products varies between 0.2 and 40.1 mg/g, compared to commercial filtered cigarettes which contain 16.3 mg/g of nicotine [7]. Their pH also varies, which, being a key determinant of the level of absorption of nicotine and carcinogens, determines its toxicity: the higher the pH, the higher the absorption and, consequently, the higher the toxicity [6]. Such considerations mean that there are substantial variations between different SLT products in the level of risk posed to human health [4, 8–11]. It is therefore important not to consider SLT as a single product, but rather as groups of products with differences in their toxicity and addictiveness depending upon their carcinogen, nicotine, and pH levels. The diversity in SLT toxicity has been an impediment not only in establishing its global risks to human health, but also in agreeing on international policies for its prevention and control. It is therefore perhaps unsurprising that despite several country-specific studies [12–15] no attempt has hitherto been made to estimate its global disease burden.

To overcome these challenges, we developed a novel approach to estimate the global burden associated with the use of SLT products. The determinants of their toxicity (carcinogens and pH) and addictiveness (nicotine) are dependent on preparation methods, ingredients that are added to SLT products, and consumption behaviours. Given that the SLT preparations and consumption patterns are determined by, and vary with, geography and culture [16], it is possible to group them according to their availability in different parts of the world (Table 1). These groups of SLT products, classified according to different geographical regions, will also be distinguishable from each other on the basis of their toxicity, addictiveness, and associated health risks. Hence, the risks were assumed to be highest in those regions and cultures where products are combined with other ingredients, and are prepared and consumed in a way that makes them very alkaline (i.e. a high pH), and rich in nicotine and TSNA [6, 17]. Building on this assumption, we aimed to estimate the worldwide burden of disease attributable to SLT use, measured in terms of disability adjusted life years (DALYs) lost and number of deaths in 2010.

## Methods

We used the comparative risk assessment method, which evaluates changes in population health (burden of disease) that result from modifying a population’s exposure to a risk factor [18, 19]. For this, we used 2010 datasets, which provided the most recent global estimates of burden of disease [20]. The estimates were calculated for individual countries and then grouped into 14 World Health Organization (WHO) sub-regions (Additional file 1: Appendix 1) [21]. These were generated through estimating the following:

1. The prevalence of SLT consumption
2. Diseases caused by SLT use
3. The relative risks of acquiring these diseases
4. The population attributable fraction (PAF) for each of these diseases
5. The overall burden of these diseases in terms of DALYs lost and deaths
6. Proportion of this burden attributable to SLT use

### Prevalence of smokeless tobacco use

We carried out a systematic literature search (see Additional file 1: Appendix 2 for a detailed description of the methods employed) for the point prevalence (current use) of SLT consumption among all adult (≥15 years) populations, and also for men and women separately. Only one prevalence report was included for one country. Latest national prevalence data collected as part of an international or regional survey were preferred over an older isolated national or a sub-national survey. We used data from the Global Adult Tobacco Survey (GATS), where available [22]. In its absence, other international (WHO STEPwise approach to Surveillance, The Demographic and Health Surveys), regional (Special Europe Barometer), national, and/or sub-national surveys were used to extract prevalence data.

### Diseases caused by smokeless tobacco use

A scoping review was carried out to identify associated diseases. A series of focused literature reviews were subsequently carried out to find and assess the evidence of causation between each of these diseases and SLT use. Our search strategies and selection criteria are provided in Additional file 1: Appendix 3. One researcher ran the searches, which were then independently scrutinised by another independent researcher who considered the search results against the pre-specified inclusion and exclusion criteria. Similarly, one researcher extracted data, which were independently crosschecked by another researcher. In particular, we appraised the studies for case definitions for diseases and for assessment methods for measuring exposure to SLT and for investigating the effects of potential confounders. We excluded those diseases (and respective studies) where evidence was not supportive of a causal relationship. Only studies that adequately controlled for smoking and/or alcohol as potential confounders either at the design or the analysis stage were carried forward into the next stage of the analysis (discussed below). Quality was assessed using the Newcastle-Ottawa Scale for assessing the quality of non-randomised studies in meta-analyses [23].

### Assessing risk and meta-analyses

Risk estimates (relative risks/odds ratios) and their confidence intervals (CI) were log transformed to produce effect sizes and standard errors, respectively [24]. We carried out random effects meta-analysis using RevMan version 5 to estimate pooled risk estimates. We first obtained country-specific risk estimates (relative risks/odds ratios) for individual diseases by pooling data from the included studies carried out in respective countries. We then extrapolated non-specific global risk estimates by pooling respective country-specific risk estimates. We were mindful that the risk of acquiring diseases varies between countries owing to differences in SLT products used. Therefore, for each disease where good country-specific risk estimates (pooled estimate from a meta-analysis of three or more studies in respective country) were available, we applied these to respective countries and also to those countries and regions where similar SLT products are used. In the absence of good country-specific risk estimates, we used either one of the following two approaches: (a) In countries and regions that use SLT products with moderate to high pH and TSNAs levels, we applied non-specific global estimates (pooled estimate from a meta-analysis of all studies); and (b) in countries and regions where there was either no information available on the SLT products or the information available indicates low levels of pH and TSNA, we did not apply any estimates. Further details on the application of these assumptions across all 14 WHO regions are provided in web Additional file 1: Appendix 4. We only used those pooled relative risks (country or non-specific) that were found to be statistically significant.

Where associations were presented for more than one SLT product in the same paper, we considered these as separate studies for the purpose of meta-analysis. Similarly, where risks were given separately for former and current SLT users, these were also treated as separate studies. We did not attempt to group risks according to gender because very few studies had such sub-group analysis.

### Population attributable fraction

PAF is the proportional reduction in disease or mortality that would occur if exposure were reduced to zero [25, 26]. PAF was estimated for each disease for each country for both males and females, using the following formula:

$$ \mathrm{P}\mathrm{A}\mathrm{F}={\mathrm{P}}_{\mathrm{e}}\left({\mathrm{RR}}_{\mathrm{e}}\hbox{--} 1\right)/\left[1+{\mathrm{P}}_{\mathrm{e}}\left({\mathrm{RR}}_{\mathrm{e}}\hbox{--} 1\right)\right] $$

$$ {\mathrm{P}}_{\mathrm{e}}=\mathrm{Prevalence} $$

$$ {\mathrm{RR}}_{\mathrm{e}}=\mathrm{Relative}\ \mathrm{Risk} $$

### Overall burden

The overall number of DALYs and deaths for each associated disease for both males and females for each country were extracted from the 2010 Global Burden of Disease study [27, 28].

### Attributable burden

The attributable burden (AB), in deaths and DALYs, was estimated for each associated disease for each country for both males and females by multiplying PAF by the overall burden of the disease (B):

$$ \mathrm{AB}=\mathrm{P}\mathrm{A}\mathrm{F}\times \mathrm{B} $$

## Results

### Prevalence of smokeless tobacco use

**Fig. 1:**
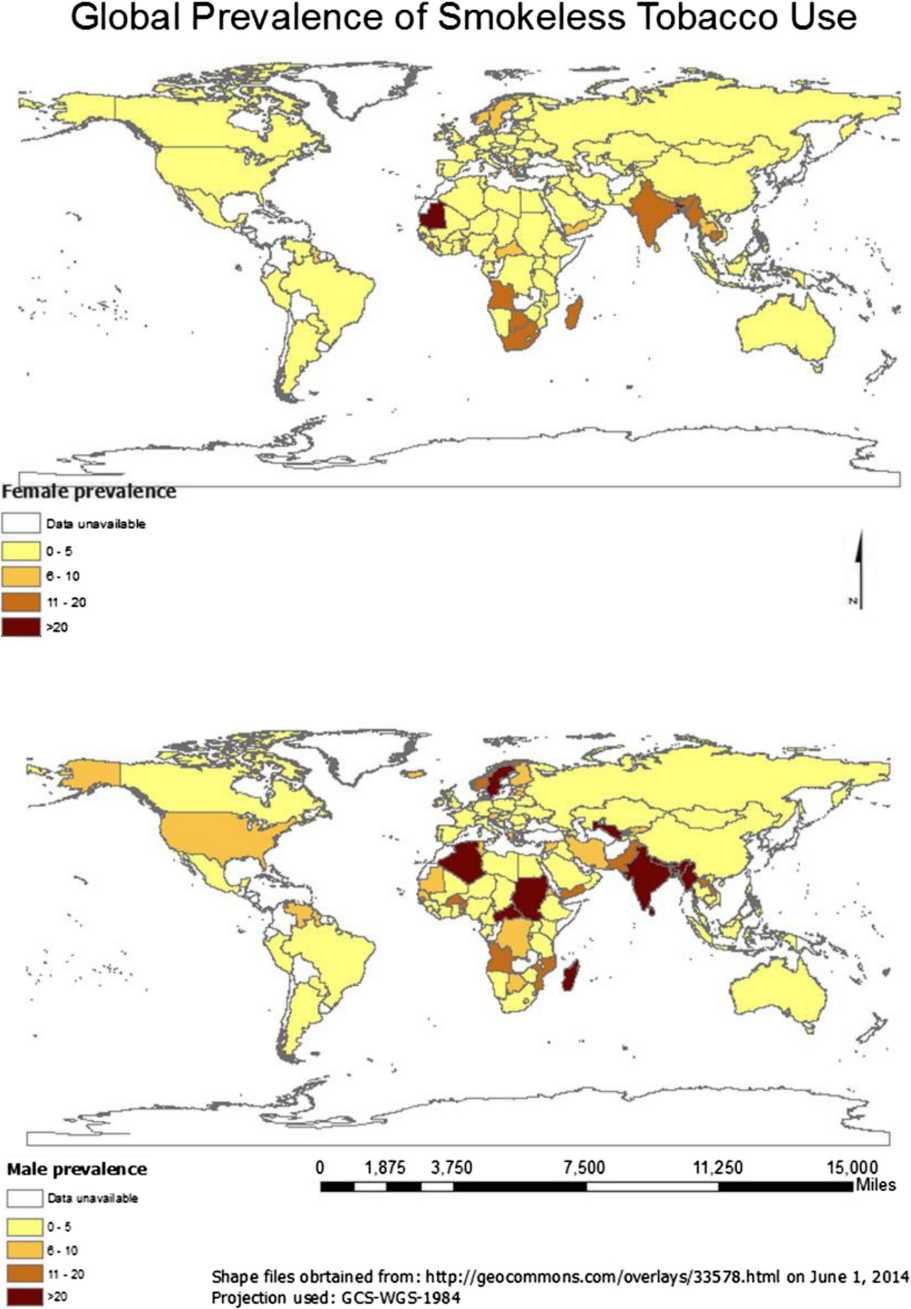
Smokeless tobacco prevalence among males and females

We found adult prevalence figures for SLT consumption in 115 countries (Fig. 1). The definition for ‘adult’ ranged from 15, 16, 25, or 35 years at one end to 49, 64, 65, 70, 74, 84, 85, 89, or no age limit at the other. The PRISMA diagram describing the selection of the prevalence reports is provided in Additional file 1: Appendix 5a.

In general, SLT consumption was higher among males than females (Table 2). Mauritania had the highest prevalence of SLT consumption among females (28.3 %), followed by Bangladesh (27.9 %), Madagascar (19.6 %), India (18.4 %), and Bhutan (17.3 %). Among males, Myanmar (51.4 %), Nepal (37.9 %), India (32.9 %), Uzbekistan (31.8 %), and Bangladesh (26.4 %) had the highest consumption rates. Within Europe, SLT (snus) consumption was high in Sweden (24.0 % males, 7.0 % females) and Norway (20.0 % males, 6.0 % females).

**Table 2:** Prevalence of smokeless tobacco use in different countries of the world according to WHO sub-regional classification

WHO sub-regions	Country	M	F	Source	Year
Africa (Region D)	Algeria	21	0.4	STEPS [38]	2005
	Benin	12.7	5.7	STEPS [38]	2008
	Burkina Faso	–	3.86	DHS [39]	2011
	Cameroon	1.94	0.94	DHS [39]	2011
	Cape Verde	3.5	5.8	STEPS [38]	2007
	Chad	1.9	0.4	STEPS [38]	2008
	Comoros	7.72	2.99	DHS [39]	2012
	Gabon	0.48	0.34	DHS [39]	2012
	Gambia	0.8	1.4	STEPS [38]	2010
	Ghana	1.33	0.2	DHS [39]	2008
	Guinea	1.4	1.5	STEPS [38]	2009
	Liberia	2.3	2.4	DHS [40]	2007
	Madagascar	24.66	19.6	DHS [39]	2009
	Mali	5	1.2	STEPS [38]	2007
	Mauritania	5.7	28.3	STEPS [38]	2006
	Niger	4.55	2.3	DHS [39]	2012
	Nigeria	3.2	0.5	DHS [40]	2008
	Sao Tome & Principe	3.8	1.9	STEPS [38]	2009
	Senegal	6.63	0.23	DHS [39]	2011
	Sierra Leone	3	12	STEPS [38]	2009
	Togo	5.1	2.2	STEPS [38]	2010
Africa (Region E)	Botswana	7.2	14.5	STEPS [38]	2007
	Burundi	0.03	0.31	DHS [39]	2011
	Congo (Brazzaville)	8.3	1.54	DHS [39]	2012
	Congo (Republic)	8.67	3.22	DHS [39]	2013
	Cote d'Ivoire	0.61	1.27	DHS [39]	2012
	Eritrea	5.8	0.2	STEPS [38]	2004
	Ethiopia	1.94	0.2	DHS [39]	2011
	Kenya	2.05	1.29	DHS [39]	2008
	Lesotho	1.3	9.1	DHS [40]	2009
	Malawi	1.9	5	STEPS [38]	2009
	Mozambique	10.94	0.82	DHS [39]	2011
	Namibia	1.8	2.3	DHS [40]	2006–07
	Rwanda	5.8	2.73	DHS [39]	2011
	South Africa	2.4	10.9	DHS [41]	2003
	Swaziland	2.6	0.8	STEPS [38]	2007
	Tanzania	2.03	0.83	DHS [39]	2010
	Uganda	2.94	1.5	DHS [39]	2011
	Zambia	0.3	1.2	DHS [39]	2007
	Zimbabwe	1.6	0.4	DHS [41]	2011
Americas (Region A)	Canada	2	–	ICS [41]	2011
	USA	6.5	0.4	ICS [41]	2010
Americas (Region B)	Argentina	0.1	0.2	GATS [42]	2012
	Barbados	0	0.6	STEPS [38]	2007
	Brazil	0.6	0.3	GATS [42]	2010
	Dominican Republic	1.9	0.3	DHS [40]	2007
	Grenada	2.2	0.3	STEPS [38]	2011
	Mexico	0.3	0.3	GATS [42]	2009
	Paraguay	3	1.6	ICS [41]	2011
	St Kitts & Nevis^a^	0.3	0.1	STEPS [38]	2007
	Trinidad & Tobago	0.5	0.3	STEPS [38]	2011
	Venezuela	6.2	0.9	ICS [41]	2011
Americas (Region D)	Haiti	–	2.5	DHS [40]	2005–06
Eastern Mediterranean (Region B)	Libya	2.2	0.1	STEPS [38]	2009
	Saudi Arabia	1.3	0.5	STEPS [38]	2004
	Tunisia	8.6	2.2	ICS [41]	2005–06
Eastern Mediterranean (Region D)	Egypt	4.8	0.3	GATS [42]	2009
	Iraq	1.6	0.3	STEPS [38]	2006
	Pakistan	16.3	2.44	DHS [43]	2012–13
	Sudan	24.1	1	STEPS [38]	2005
	Yemen	15.1	6.2	ICS [41]	2003
Europe (Region A)	Austria	7.8	1.1	SEBS [44]	2012
	Belgium	1.1	0.6	SEBS [44]	2012
	Cyprus	2.1	0.4	SEBS [44]	2012
	Czech Republic	2.5	0.4	SEBS [44]	2012
	Denmark	3	1	ICS [41]	2010
	Finland	5.5	0.3	ICS [41]	2011
	France	1.2	0.6	SEBS [44]	2012
	Germany	3.4	3.4	SEBS [44]	2012
	Iceland	5.97	–	ICS [41]	2008
	Ireland	2.2	0.9	SEBS [44]	2012
	Italy	1.8	1.5	SEBS [44]	2012
	Luxembourg	1.8	1	SEBS [44]	2012
	Malta	5.5	1.5	SEBS [44]	2012
	Netherlands	0.3	0.1	ICS [41]	2011
	Norway	20	6	ICS [41]	2011
	Portugal	4.4	1.1	SEBS [44]	2012
	Slovenia	1.8	0.4	SEBS [44]	2012
	Spain	0.4	0.2	SEBS [44]	2012
	Sweden	24	7	ICS [41]	2011
	Switzerland	4	1.3	ICS [41]	2011
	United Kingdom	1.6	0.5	SEBS [44]	2012
Europe (Region B)	Ajerbaijan	0.3	0	DHS [40]	2006
	Armenia	1.8	0	DHS [40]	2005
	Bulgaria	0.3	0	SEBS [44]	2012
	Georgia	1	0.2	ICS [41]	2010
	Kyrgyzstan	7	0.3	ICS [41]	2006
	Poland	1	0.1	GATS [42]	2009
	Romania	0.4	0.2	GATS [42]	2011
	Slovakia	3.9	0.7	SEBS [44]	2012
	Uzbekistan	31.8	0.2	DHS [40]	2002
Europe (Region C)	Latvia	5.8	0.9	ICS [41]	2010
	Lithuania	1.2	0.2	SEBS [44]	2012
	Moldova	0.1	0	DHS [40]	2005
	Russia	1	0.2	GATS [42]	2009
	Ukraine	0.5	0	GATS [42]	2010
South East Asia (Region B)	Indonesia	1.5	2	GATS [42]	2011
	Sri Lanka	24.9	6.9	STEPS [38]	2006
	Thailand	1.1	5.2	GATS [42]	2011
South East Asia (Region D)	Bangladesh	26.4	27.9	GATS [42]	2009
	Bhutan	21.1	17.3	STEPS [38]	2007
	India	32.9	18.4	GATS [42]	2009
	Maldives	5.6	2.6	STEPS [38]	2011
	Myanmar	51.4	16.1	STEPS [38]	2009
	Nepal	37.9	6	DHS [41]	2011
	Timor Leste	2.48	1.93	DHS [43]	2009–10
Western Pacific (Region A)	Australia	0.75	0.41	ICS [45]	2004
Western Pacific (Region B)	Cambodia	2.2	14.8	STEPS [38]	2010
	China	0.7	0	GATS [42]	2010
	Lao People’s Democratic Republic	14.6	1.1	STEPS [38]	2008
	Malaysia	0.9	0.6	GATS [42]	2011
	Micronesia	22.4	3	STEPS [38]	2002
	Mongolia	2.8	0.5	STEPS [38]	2009
	Philippines	2.8	1.2	GATS [42]	2009
	Vietnam	0.3	2.3	GATS [42]	2010

*DHS* The Demographic and Health Surveys, *ICS* Individual Country Survey, *GATS* Global Adult Tobacco Survey, *SEBS* The Special Europe Barometer Survey, *STEPS* STEPwise approach to Surveillance

^a^Populations of St Kitts and Nevis are tiny and unlikely to affect our estimates

### Diseases caused by smokeless tobacco use

**Fig. 2:**
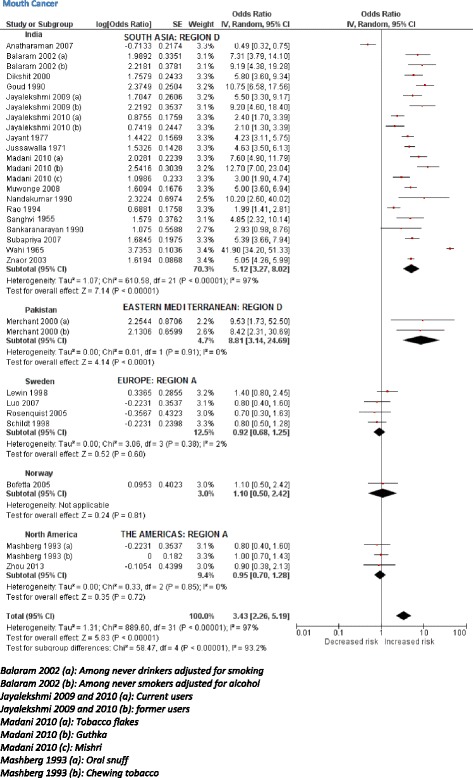
Random effects model showing relative risk for mouth cancer for smokeless tobacco use

**Fig. 3:**
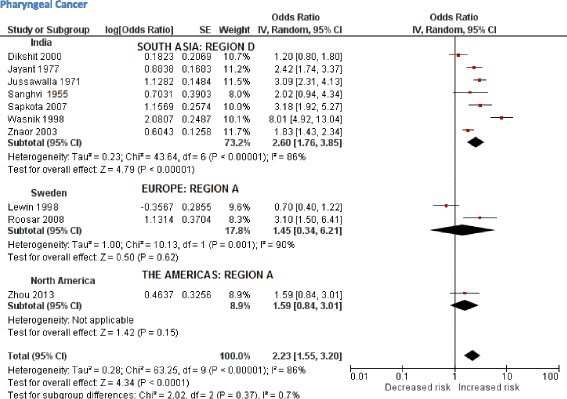
Random effects model showing relative risk for pharyngeal cancer for smokeless tobacco use

**Fig. 4:**
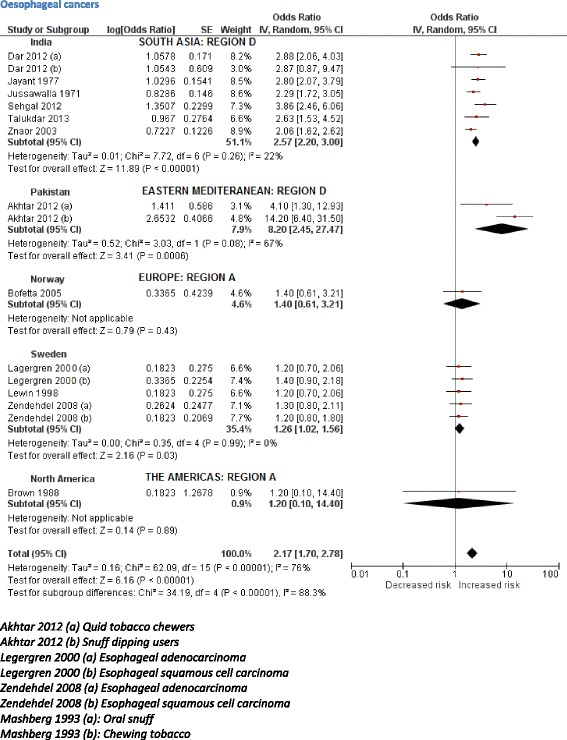
Random effects model showing relative risk for oesophageal cancer for smokeless tobacco use

**Fig. 5:**
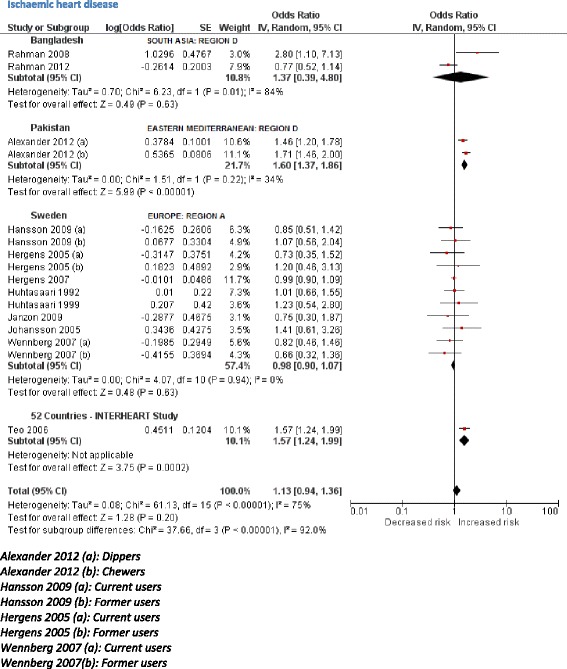
Random effects model showing relative risk for ischaemic heart disease for smokeless tobacco use

The initial scoping review identified a number of associated diseases, including a range of cancers, cardiovascular diseases (ischaemic heart disease and stroke), periodontal conditions, and adverse pregnancy outcomes. The subsequent more focused systematic reviews identified 53 studies (Table 3) reporting association between SLT consumption and cancers of mouth, pharynx, larynx, oesophagus, lung, and pancreas (39 studies); and cardiovascular diseases, such as ischaemic heart disease and stroke (14 studies). PRISMA flow diagrams describing the selection process of the studies identified in the literature searches are provided in Additional file 1: Appendix 5b,c. The pooled non-specific relative risks were statistically significant for cancers of the mouth, pharynx, and oesophagus (Figs. 2, 3, 4, and 5). Only statistically significant relative risks (country*-*specific or non-specific) were included in the model to estimate attributable risks. For example, the pooled non-specific relative risk for laryngeal cancer was 1.42 (95 % CI 0.77–2.59), and hence excluded (Additional file 1: Appendix 6). Likewise, none of the country-specific estimates for the USA were statistically significant (Additional file 1: Appendix 4). Based on the above reviews, we assumed that a causal association exists between some SLT products and cancers of the mouth, pharynx, and oesophagus, and ischaemic heart disease.

**Table 3:** Smokeless tobacco use and risk of cancers, ischaemic heart disease, and stroke—studies included in meta-analysis

Country	Study period	Study design	Exposure status	Inclusion of cigarette/alcohol users	Outcome	Odds ratios/relative risks (95 % confidence intervals)	Comments	Quality assessment (NOS)^a^	Reference
CANCERS									
India	2001–2004	Case–control	Smokeless tobacco with or without additives	No/No	Oral cancer	0.49 (0.32–0.75)	Exclusive SLT users	Selection****	Anantharaman et al. 2007 [46]
								Comparability**	
								Exposure/Outcome*	
India	1996–1999	Case–control	Ever SLT users	Yes/Yes	Oral cancer	7.31 (3.79–14.1)	Never drinkers adjusted for smoking	Selection****	Balaram et al. 2002 [47]
						9.19 (4.38–19.28)	Never smokers adjusted for alcohol	Comparability**	
								Exposure/Outcome *	
India	1982–1992	Case–control	Tobacco quid chewing	Yes/No	Oral cancer	5.8 (3.6–9.34)	Adjusted for smoking	Selection***	Dikshit & Kanhere 2000 [48]
					Pharyngeal cancer	1.2 (0.8–1.8)		Comparability*	
					Lung cancer	0.7 (0.4–1.22)		Exposure/Outcome*	
India	Unclear	Case–control	Chewing tobacco	No/No	Oral cancer	10.75 (6.58–17.56)	Exclusive SLT users	Selection**	Goud et al. 1990 [49]
								Comparability*	
								Exposure/Outcome^0^	
India	1990–1997	Cohort	Current SLT users	No/No	Oral cancer	5.5 (3.3–9.17)	Exclusive SLT users	Selection****	Jayalekshmi et al. 2009 [50]
			Former SLT users			9.2 (4.6–18.40)		Comparability*	
								Exposure/Outcome**	
India	1990–1997	Cohort	Current SLT user	Yes/Yes	Oral cancer	2.4 (1.7–3.39)	Adjusted for smoking and alcohol	Selection****	Jayalekshmi et al. 2010 [51]
			Former SLT users			2.1 (1.3–3.39)		Comparability*	
								Exposure/Outcome***	
India	May 2005	Case–control	Ever SLT users	No/No	Oral cancer	4.23 (3.11–5.75)	Exclusive SLT users	Selection***	Jayant et al. 1977 [52]
					Pharyngeal cancer	2.42 (1.74–3.37)		Comparability**	
					Laryngeal cancer	2.8 (2.07–3.79)		Exposure/Outcome^0^	
					Oesophageal cancer	1.55 (1.15–2.07)			
India	1968	Case–control	Tobacco	Yes/No	Oral cancer	4.63 (3.50–6.14)	Exclusive chewers and non-chewers data available	Selection***	Jussawalla & Deshpande 1971 [53]
					Pharyngeal cancer	3.09 (2.31–4.13)		Comparability**	
					Laryngeal cancer	2.29 (1.72–3.05)		Exposure/Outcome^0^	
					Oesophageal cancer	3.82 (2.84–5.13)			
India	2005–2006	Case–control	Tobacco flakes	Yes/Yes	Oral cancer	7.6 (4.9–11.79)	Adjusted for smoking and alcohol	Selection****	Madani et al. 2010 [54]
			Gutkha			12.7 (7–23.04)		Comparability**	
			Mishiri			3.0 (1.9–4.74)		Exposure/Outcome*	
India	Unclear	Case–control	Chewing tobacco	Yes/Yes	Oral cancer	5.0 (3.6–6.94)	Adjusted for smoking and alcohol	Selection****	Muwonge et al. 2008 [55]
								Comparability*	
								Exposure/Outcome*	
India	1982–1984	Case–control	Chewing tobacco	Yes/No	Oral cancer	10.2 (2.6–40.02)	Adjusted for smoking	Selection***	Nandakumar et al. 1990 [56]
								Comparability**	
								Exposure/Outcome*	
India	1980–1984	Case–control	SLT users	No/No	Oral cancer	1.99 (1.41–2.81)	Exclusive SLT users	Selection**	Rao et al. 1994 [57]
								Comparability^0^	
								Exposure/Outcome*	
India	1952–1954	Case–control	Chewing tobacco	No/No	Oral cancer	4.85 (2.32–10.14)	Exclusive SLT users	Selection***	Sanghvi et al. 1955 [58]
					Pharyngeal cancer	2.02 (0.94–4.33)		Comparability**	
					Laryngeal cancer	0.76 (0.37–1.56)		Exposure/Outcome^0^	
India	1983–1984	Case–control	Snuff (males only)	Yes/Yes	Oral cancer	2.93 (0.98–8.76)	Adjusted for smoking and alcohol; adjusted effect size is only among males	Selection***	Sankaranarayan et al. 1990 [59]
								Comparability^0^	
								Exposure/Outcome*	
India	Not given	Case–control	Tobacco chewing	Yes/Yes	Oropharyngeal cancer	7.98 (4.11–13.58)^b^	Adjusted for smoking and alcohol	Selection***	Wasnik et al. 1998 [60]
								Comparability**	
								Exposure/Outcome^0^	
India	1991–2003	Case–control	Chewing tobacco	No/No	Oral cancer	5.88 (3.66–7.93)	Exclusive SLT users	Selection****	Subapriya e al. 2007 [61]
								Comparability**	
								Exposure/Outcome**	
India	1950–1962	Case–control	Tobacco with or without paan or lime	Yes/No	Oral and oropharyngeal cancer	41.90 (34.20–51.33)	Exclusive chewer data available	Selection**	Wahi et al. 1965 [62]
							Note: data of habit was not available for the whole cohort	Comparability**	
								Exposure/Outcome^0^	
Pakistan	1996–1998	Case–control	Naswar	Yes/Yes	Oral cancer	9.53 (1.73–52.50)	Adjusted for smoking and alcohol	Selection***	Merchant et al. 2000 [63]
			Paan with tobacco			8.42 (2.31–30.69)		Comparability**	
								Exposure/Outcome*	
Sweden	1973–2002	Cohort	Snus	Yes/Yes	Oral and pharyngeal combined	3.10 (1.50–6.41)	Adjusted for smoking and alcohol	Selection**	Roosar et al. 2008 [64]
								Comparability**	
								Outcome***	
India	1993–1999	Case–control	Chewing tobacco	Yes/Yes	Oral cancer	5.05 (4.26–5.99)	Adjusted for smoking and alcohol	Selection***	Znaor et al. 2003 [65]
					Pharynx	1.83 (1.43–2.34)		Comparability**	
					Oesophagus	2.06 (1.62–2.62)		Exposure/Outcome*	
Norway	1966–2001	Cohort	Chewing tobacco plus oral snuff	No/No	Oral cancer	1.1 (0.5–2.42)	Adjusted for smoking, might be confounded by alcohol use	Selection***	Bofetta et al. 2005 [66]
					Oesophageal cancer	1.4 (0.61–3.21)		Comparability*	
					Pancreatic cancer	1.67 (1.12–2.49)		Exposure/Outcome***	
					Lung cancer	0.80 (0.61–1.05)			
Sweden	1988–1991	Case–control	Oral snuff	Yes/Yes	Oral cancer	1.4 (0.8–2.45)	Adjusted for smoking and alcohol	Selection**	Lewin et al. 1998 [67]
					Larynx	0.9 (0.5–1.62)		Comparability**	
					Oesophagus	1.2 (0.7–2.06)		Exposure/Outcome*	
					Pharynx	0.7 (0.4–1.22)			
Sweden	1969–1992	Cohort	Snus	No/No	Oral cancer	0.8 (0.4–1.60)	Exclusive SLT users	Selection***	Luo et al. 2007 [68]
					Lung cancer	0.8 (0.5–1.28)		Comparability*	
					Pancreatic cancer	2 (1.20–3.33)		Exposure/Outcome***	
Sweden	2000–2004	Case–control	Oral snuff	Yes/Yes	Oral	0.70 (0.3–1.63)	Adjusted for smoking and alcohol	Selection***	Rosenquist et al 2005 [69]
								Comparability**	
								Exposure/Outcome**	
Sweden	1980–1989	Case–control	Oral snuff	Yes/Yes	Oral cancer	0.8 (0.5–1.28)	Adjusted for smoking and alcohol	Selection**	Schildt et al. 1998 [70]
								Comparability**	
								Exposure/Outcome***	
USA	1972–1983	Case–control	Oral snuff	Yes/Yes	Oral cancer	0.8 (0.4–1.60)	Not clear if adjusted for smoking and alcohol	Selection**	Mashberg et al. 1993 [71]
			Chewing tobacco			1 (0.7–1.43)		Comparability^0^	
								Exposure/Outcome*	
USA	Not given	Case–control	SLT use	Yes/Yes	Oral cancer	0.90 (0.38–2.13)	Adjusted for smoking and alcohol	Selection***	Zhou et al. 2013 [15]
					Pharyngeal cancer	1.59 (0.84–3.01)		Comparability**	
					Laryngeal cancer	0.67 (0.19–2.36)		Exposure/Outcome*	
India	2001–2004	Case–control	Chewing tobacco	No/No	Pharyngeal cancer	3.18 (1.92–5.27)	Exclusive SLT users	Selection***	Sapkota et al. 2007 [72]
					Laryngeal cancer	0.95 (0.52–1.74)		Comparability**	
								Exposure/Outcome*	
Pakistan	1998–2002	Case–control	Snuff dipping	No/No	Oesophageal cancer	4.1 (1.3–12.93)	Adjusted for areca nut	Selection***	Akhtar et al. 2012 [73]
			Quid with tobacco			14.2 (6.4–31.50)		Comparability**	
								Exposure/Outcome**	
India	2008–2012	Case–control	Nass chewing	No/No	Oesophageal cancer	2.88 (2.06–4.03)	Exclusive SLT users	Selection***	Dar et al. 2012 [74]
			Gutkha chewing			2.87 (0.87–9.47)		Comparability**	
								Exposure/Outcome**	
India	2007–2011	Case–control	Oral snuff	Yes/Yes	Oesophageal cancer	3.86 (2.46–6.06)	Adjusted for smoking and alcohol	Selection**	Sehgal et al. 2012 [75]
								Comparability**	
								Exposure/Outcome*	
India	2011–2012	Case–control	Chewing tobacco	Yes/Yes	Oesophageal cancer	2.63 (1.53–4.52)	Adjusted for smoking and alcohol	Selection***	Talukdar et al. 2013 [76]
								Comparability**	
								Exposure/Outcome*	
Sweden	1995–1997	Case–control	Oral snuff	Yes/Yes	Oesophageal cancer (adenocarcinoma)	1.2 (0.7–2.06)	Adjusted for smoking and alcohol	Selection***	Lagergren et al. 2000 [77]
					(Squamous cell carcinoma)	1.4 (0.9–2.18)		Comparability**	
								Exposure/Outcome*	
Sweden	1969–1993	Cohort	Oral snuff	Yes/No	Oesophageal cancer (Adenocarcinoma)	1.3 (0.8–2.11)	Adjusted for smoking	Selection**	Zendehdel et al. 2008 [78]
					(Squamous cell carcinoma)	1.2 (0.8–1.80)		Comparability*	
								Exposure/Outcome**	
Sweden	1974–1985	Cohort	SLT users	No/NA	Lung cancer	0.90 (0.20– 4.05)	Adjusted for age, region of origin	Selection***	Bolinder et al. 1994 [79]
								Comparability*	
								Outcome**	
Morocco	1996–1998	Case–control	SLT users	Yes/No	Lung cancer	1.05 (0.28–3.94)	Adjusted for smoking	Selection**	Sasco et al. 2002 [80]
								Comparability**	
								Exposure/Outcome**	
USA	1977–1984	Case–control	SLT users	Yes/No	Oesophageal cancer	1.2 (0.1–14.40)	Adjusted for smoking	Selection***	Brown et al. 1988 [81]
								Comparability**	
								Exposure/Outcome**	
USA	1986–1989	Case–control	SLT users	Yes/No	Pancreatic cancer	1.4 (0.5–3.92)	Adjusted for smoking	Selection***	Alguacil & Silverman 2004 [82]
								Comparability*	
								Exposure/Outcome**	
USA	2000–2006	Case–control	Chewing tobacco	Yes/Yes	Pancreatic cancer	0.6 (0.3–1.20)	Adjusted for smoking and alcohol	Selection****	Hassan et al. 2007 [83]
			Oral snuff			0.5 (0.1–2.5)		Comparability**	
								Exposure/Outcome*	
CARDIOVASCULAR DISEASES (ischaemic heart disease and stroke)									
52 countries	1999–2003	Case–control	Chewing tobacco	No/Yes	Myocardial infarction	1.57 (1.24–1.99)	Adjusted for diabetes, abdominal obesity, hypertension, exercise, diet	Selection****	Teo et al. 2006 [29]
								Comparability**	
								Exposure/Outcome*	
Pakistan	2005–2011	Case–control	Dippers only (Naswar)	No/NA	Myocardial infarction	1.46 (1.20–1.77)	Adjusted for age, sex, region, ethnicity	Selection****	Alexander 2013 [84]
			Chewers only (Paan/ Supari/ Gutkha)			1.71 (1.46–2.00)		Comparability**	
								Exposure/Outcome**	
Bangladesh	2006–2007	Case–control	Ever SLT users	No/NA	Myocardial infarction, Angina pectoris	2.8 (1.1–7.13)	Adjusted for age, sex, hypertension	Selection***	Rahman & Zaman 2008 [85]
								Comparability**	
								Exposure/Outcome*	
Bangladesh	2010	Case–control	Ever SLT users	No/NA	Myocardial infarction, Angina pectoris	0.77 (0.52–1.14)	Adjusted for age, hypertension, diabetes, acute psycho-social stress	Selection****	Rahman et al. 2012 [86]
								Comparability**	
								Exposure/Outcome*	
Sweden	1998–2005	Case–control	Current SLT users	No/NA	Myocardial infarction	0.73 (0.35–1.52)	Exclusive SLT users	Selection***	Hergens et al. 2005 [87]
			Former SLT users			1.2 (0.46–3.13)		Comparability**	
								Exposure/Outcome**	
Sweden	1978–2004	Cohort	Ever SLT users	No/NA	Myocardial infarction	0.99 (0.90–1.10)	Adjusted for age, BMI, region of residence	Selection**	Hergens et al. 2007 [88]
								Comparability**	
								Exposure/Outcome***	
Sweden	1989–1991	Case–control	Regular SLT users	Yes/NA	Myocardial infarction	1.01 (0.66–1.55)^c^	Adjusted for age, education, smoking	Selection***	Huhtasaari et al. 1992 [89]
								Comparability**	
								Exposure/Outcome*	
Sweden	1991–1993	Case–control	Former SLT users	No/NA	Myocardial infarction	1.23 (0.54–2.82)	Exclusive SLT users	Selection****	Huhtasaari et al. 1999 [90]
								Comparability**	
								Exposure/Outcome**	
Sweden	1988–2000	Cohort	Daily SLT users	No/NA	Ischaemic heart disease	1.41 (0.61–3.28)	Adjusted for BMI, physical activity, diabetes, hypertension	Selection****	Johansson et al. 2005 [91]
								Comparability**	
								Exposure/Outcome**	
Sweden	1985–1999	Case–control	Current SLT users	No/NA	Myocardial infarction	0.82 (0.46–1.46)	Adjusted for BMI, physical activity, education, cholesterol	Selection****	Wennberg et al. 2007 [92]
			Former SLT users			0.66 (0.32–1.36)		Comparability**	
								Exposure/Outcome**	
Sweden	1985–2000	Case–control	Regular SLT users	No/NA	Stroke	0.87 (0.41–1.83)	Adjusted for diabetes, hypertension, education, marital status, cholesterol	Selection****	Asplund et al. 2003 [93]
								Comparability**	
								Exposure/Outcome**	
Sweden	1978–2003	Cohort	Ever SLT users	No/NA	Stroke	1.02 (0.92–1.13)	Adjusted for age, BMI, region of residence	Selection**	Hergens et al. 2008 [94]
								Comparability**	
								Exposure/Outcome***	
Sweden	1998–2005	Cohort	Current SLT users	No/NA	Ischaemic heart disease	0.85 (0.51–1.42)	Adjusted for age, hypertension, diabetes, cholesterol	Selection***	Hansson et al. 2009 [95]
			Former SLT users		Stroke	1.07 (0.56–2.04)		Comparability**	
						1.18 (0.67–2.08)		Exposure/Outcome**	
						1.35 (0.65–2.82)			
Sweden	1991–2004	Cohort	SLT users	No/NA	Myocardial infarction	0.75 (0.3–1.87)	Adjusted for age, diabetes, occupation, hypertension, physical activity, BMI, marital status	Selection***	Janzon et al. 2009 [96]
					Stroke	0.59 (0.2–1.5)		Comparability**	
								Exposure/Outcome**	

*BMI* body mass index, *NA* not applicable, *NOS* Newcastle-Ottawa Scale, *SLT* smokeless tobacco

^a^NOS for assessing the quality of non-randomised studies in meta-analyses based on selection, comparability, and exposure/outcome. Number of stars (*) indicates the number of criteria met for each of these three categories [23]

^b^Effect sizes are for oral and pharyngeal cancers combined and were included in the meta-analysis for oral cancer only

^c^Based on parameter estimate and standard error reported in paper

### Relative risks

Based on 32 studies, the estimated pooled non-specific relative risk for mouth (oral cavity, tongue, and lip) cancers was 3.43 (95 % CI 2.26–5.19) (Fig. 2). Studies from South-East Asia indicated an increased risk of oral cancer for SLT use whereas results from studies pertaining to Europe and the Americas did not substantiate such an association. For cancers of the pharynx, pooled non-specific relative risk was 2.23 (95 % CI 1.55–3.20), based on ten studies (Fig. 3). For oesophageal cancers, no clear increased risk was present in studies in the USA, whereas a pooled estimate reported a relative risk of 2.17 (95 % CI 1.70–2.78) (Fig. 4). For ischaemic heart disease, no good country-specific risk estimates were available (Fig. 5). However, we found one large case–control study (INTERHEART study) [29] conducted in 52 countries from all regions showing a statistically significant risk of ischaemic heart disease (adjusted odds ratio 1.57, 95 % CI 1.24–1.99) among SLT users.

### Applying risk estimates

For cancers in general, pooled country-specific risk estimates obtained from Sweden and the USA were applied to Europe A and Americas A, respectively. For South-East Asia B and D and Western Pacific B regions, country-specific estimates from India were applied. There were a few exceptions to this rule, because some countries (UK, Mexico, Pakistan, China, Mongolia) differed in their SLT consumption patterns from their respective regions (see Additional file 1: Appendix 4 for details). In short, country-specific risk estimates for cancers could only be fully applied to five regions. For the remaining nine regions, our findings were imputed either by applying statistically significant non-specific risk estimates or none at all (Additional file 1: Appendix 4). In case of ischaemic heart disease, Sweden was the only country with a pooled country-specific relative risk (0.98, 95 % CI 0.90–1.07) obtained from a good number (more than three) of studies. For 11 out of 14 regions, we used a large multi-country study (INTERHEART)—conducted in 52 countries—to apply and deduce risk estimates. The three regions (Europe A and C and Americas D) were excluded, as these were not among those regions included in the INTERHEART study (Additional file 1: Appendix 4). There was one exception (UK) where INTERHEART study estimates were applied because SLT products consumed in the UK commonly originate from South Asia.

### Attributable burden

The attributable burden of SLT use is outlined in Table 4. Our estimates indicate that in 2010, SLT use led to 1,711,539 DALYs lost and 62,283 deaths due to cancers of mouth, pharynx, and oesophagus, and, based on data from the benchmark 52 country INTERHEART study, 4,725,381 DALYs lost and 204,309 deaths from ischaemic heart disease. In total, SLT use caused the loss of 6,436,920 DALYs and 266,592 deaths. The figures show that three-quarters of these deaths and loss of DALYs were among males. This disease burden was found to be distributed across all WHO sub-regions. However, nearly 85 % of the total burden attributable to SLT use was in South-East Asia, with India alone accounting for 74 % of the global burden, followed by Bangladesh (5 %).

**Table 4:** Number of DALYs lost and deaths from SLT use in 2010, by WHO sub-region as defined in Additional file 1: Appendix 1

WHO sub-regions^a^	Mouth cancer			Pharyngeal cancer			Oesophageal cancer			Ischaemic heart disease			All causes		
	M	F	All	M	F	All	M	F	All	M	F	All	M	F	All
DEATHS															
Africa D	86	36	123	15	2	17	157	77	233	2323	751	3074	2581	866	3448
Africa E	155	85	240	19	12	31	389	252	641	1202	923	2125	1765	1272	3037
Americas A	0	0	0	0	0	0	0	0	0	10,240	649	10,889	10,240	649	10,889
Americas B	90	11	102	28	3	31	74	9	83	1030	291	1321	1222	314	1536
Americas D	0	0	0	0	0	0	0	0	0	0	0	0	0	0	0
Eastern Mediterranean B	11	1	12	1	0	2	4	1	5	441	74	515	457	76	534
Eastern Mediterranean D	933	254	1187	604	59	663	1012	129	1141	7401	926	8327	9950	1368	11,318
Europe A	66	13	78	16	2	18	244	38	282	539	145	684	865	197	1062
Europe B	146	3	148	57	1	58	260	2	262	5506	156	5662	5969	162	6130
Europe C	0	0	0	0	0	0	0	0	0	0	0	0	0	0	0
South-East Asia B	438	396	835	129	58	187	243	139	382	3205	1852	5057	4016	2445	6461
South-East Asia D	11,527	6459	17,987	12,715	3485	16,200	15,247	5625	20,873	117,523	45,047	162,570	157,013	60,617	217,630
Western Pacific A	0	0	0	0	0	0	0	0	0	69	36	104	69	36	104
Western Pacific B	134	159	293	22	34	56	51	63	114	3167	814	3981	3374	1070	4443
Worldwide	13,586	7418	21,003	13,608	3656	17,264	17,680	6336	24,016	152,647	51,662	204,309	197,520	69,072	266,592
DALYs															
Africa D	2516	1046	3562	452	65	517	4119	1906	6024	64,043	19,116	83,159	71,130	22,132	93,262
Africa E	4926	2293	7220	573	349	922	10,159	6290	16,449	33,502	21,109	54,610	49,159	30,042	79,201
Americas A	0	0	0	0	0	0	0	0	0	172,206	7213	179,419	172,206	7213	179,419
Americas B	2311	230	2541	734	63	797	1717	176	1893	22,252	4728	26,980	27,014	5197	32,210
Americas D	0	0	0	0	0	0	0	0	0	0	0	0	0	0	0
Eastern Mediterranean B	285	36	321	33	9	43	86	20	106	9841	1383	11,224	10,246	1448	11,694
Eastern Mediterranean D	29,240	7669	36,909	16,446	1800	18,247	27,777	3613	31,390	187,394	21,544	208,938	260,857	34,627	295,483
Europe A	1514	224	1738	369	45	414	4949	545	5494	8397	1491	9888	15,230	2304	17,534
Europe B	4439	60	4499	1704	20	1724	6460	56	6517	115,640	1991	117,631	128,243	2128	130,371
Europe C	0	0	0	0	0	0	0	0	0	0	0	0	0	0	0
South-East Asia B	10,968	7741	18,709	3217	1487	4704	5608	2983	8591	66,969	29,913	96,881	86,762	42,124	128,886
South-East Asia D	351,752	179,051	530,803	338,976	107,041	446,017	400,770	143,146	543,916	290,6993	938,528	3,845,521	3,998,491	1,367,766	5,366,257
Western Pacific A	0	0	0	0	0	0	0	0	0	1024	340	1364	1024	340	1364
Western Pacific B	3700	3567	7267	615	794	1409	1313	1485	2797	72,936	16,830	89,766	78,564	22,675	101,239
Worldwide	411,652	201,918	613,569	363,120	111,673	474,793	462,957	160,219	623,177	3,661,195	1,064,186	4,725,381	4,898,924	1,537,996	6,436,920

## Discussion

We have found that SLT is consumed worldwide and that its use results in substantial, potentially avoidable, morbidity and mortality. However, owing to marked differences in the types of products available, patterns of consumption, and associated risks, there are substantial differences in the attributable burden between regions and countries. In particular, SLT consumption in South-East Asia leads to a much greater burden of disease than in Sweden, despite its use being equally prevalent. This is due to the much lower levels of TSNA and pH in SLT products in Sweden compared to those found in SLT in South-East Asia [6]. Similarly, SLT products used in the USA have lower risk estimates than for those used in South-East Asia.

We found that more than six million DALYs were lost and over a quarter of a million deaths occurred in 2010 owing to SLT consumption. However, our estimates require cautious interpretation because of a number of potential limitations.

First, our analysis was limited to those countries and diseases for which reliable prevalence and risk data were available, respectively. Most global tobacco surveys that reported on SLT consumption did not include all countries in the world. While global figures on smoking prevalence were available, we did not find any SLT prevalence figures for almost half of all countries. Where SLT prevalence figures were available, two countries (Micronesia and Saint Kitts & Nevis) were excluded from the final estimates owing to an absence of data for cancers in the 2010 Global Burden of Disease study. Moreover, for certain disease outcomes, e.g. adverse reproductive and oral health effects, poor quality as well as limited quantity of evidence precluded their inclusion.

Second, lack of country-specific risk estimates leads to considerable uncertainty. Despite several countries reporting SLT consumption, most did not have any reliable information on the types of SLT products used and on their associated health risks. For example, studies from several African countries reported high SLT consumption (Table 2), but provided little information on their hazard profile. There is some evidence, mainly from Sudan [30], that products used in Africa tend to have a higher pH than those used in Europe or in the USA. However, we did not find any data on the risks associated with widespread SLT use in southern parts of Africa. Likewise, various forms of SLT have been used in parts of South America (Brazilian rapê or Venezuelan chimó) for many years, yet there are no studies on the health effects of such products. In the absence of country-specific risk estimates, we assumed that in general those populations that consume similar SLT products are likely to share similar health risks and susceptibilities. We extrapolated and applied risk estimates to most countries included in our analysis on that basis (Additional file 1: Appendix 4). For cancer, our extrapolation was based on estimates obtained from several studies; for ischaemic heart diseases, extrapolations were mostly based on a single although large multi-country study (INTERHEART). As a result, almost three-quarters of the estimated SLT disease burden, which is attributed to ischaemic heart disease, is uncertain. Therefore, a cautious interpretation would be to exclude ischaemic heart disease burden figures from our estimates. However, in estimating these figures we had already excluded those regions and their respective countries that were not included in INTERHEART study. As a pointer on future research, our study highlights the need to study risk of SLT consumption on ischaemic heart diseases across the spectrum of SLT products and consumption behaviours. In time, this will produce more country-specific risk estimates, which would undoubtedly improve the reliability of our estimates presented here.

Third, the disease burden observed in 2010 is unlikely to be a consequence of SLT consumption in recent years. Therefore, our prevalence figures, obtained in surveys carried out in the last decade and used in the estimates, could be problematic. However, we assumed that the SLT consumption rates have remained stable over the last 30–40 years in these countries. We consider this as a safe assumption given that SLT use is not a new trend and historically embedded in culture and tradition in many countries, most remarkably in South Asia [31]. Consumption trends based on repeated youth surveys in India and Bangladesh suggest that SLT use has remained stable over the last decade [32]. Evidence from Sweden suggests that while more people are using snus now than 25 years ago, the consumption trends, compared to cigarette use, have essentially remained stable in this period [33, 34].

Finally, the age range of the adult sampling frames used in different SLT prevalence surveys varied, which could also increase uncertainty. The main difference between two of the key categories used was in the adult range starting from either ≥15 years or ≥25 years. Given that the risk of cancers and ischemic heart disease accumulates after many years of use well beyond young adult age, it may not have made much of a difference to our burden of disease estimates.

For the seven countries in South-East Asia region D, we estimated that 55,060 deaths caused by cancers of mouth, pharynx, and oesophagus, could be attributed to SLT in 2010. This is a little higher than the estimates from a recent study in which 50,000 deaths were attributed to SLT in eight South Asian countries [4]. This discrepancy may be explained by the fact that we used the most recent, updated prevalence and burden of disease figures.

Our estimate does not include economic impact. However, given the nature of the associated diseases, it is likely that the SLT use imposes a huge economic burden on weak health systems and poor economies. Moreover, owing to higher consumption of SLT among people of lower socio-economic status and inequitable access to health care in low-income and middle-income countries, its use is likely to contribute to driving disadvantaged sections of these societies into further poverty. A disproportionate impact on the male population (more than 70 % of disease burden due to SLT is in males) is also likely to have a disproportionate economic impact on societies in terms of reduced workforce contributions by men. On the other hand, effective legislation, policy, and preventive programmes could avert this burden due to SLT.

The signatories of the WHO’s Framework Convention on Tobacco Control should, in addition to the focus on reducing smoking consumption and related harm, now also consider the need to regulate production, marketing, and labelling of SLT products. This is particularly necessary in those countries where prevalence is high and SLT products are manufactured at a large scale without any checks on the carcinogenic level of their ingredients [35]. In countries where its use is largely limited to immigrant populations (such as in the UK) [36], strict regulation and taxation policies should be enforced which prevent import of SLT products and sale by local shops.

SLT is an important health issue, applying to a large part of the world. The data presented here are the most comprehensive gathered and brought together thus far. However, considerable uncertainties remain pertaining to risk estimation of different diseases associated with SLT use. Therefore more research is needed to investigate the newly established and previously known adverse health outcomes pertaining to SLT, particularly within countries where prevalence is high but no research evidence of risk estimation is available. Moreover, more descriptive questions about the type of SLT products and the pattern of use should be introduced into national surveys and publications of such findings encompassing all the regions.

## Conclusions

Our study, a first attempt to assess global burden of disease due to SLT, estimates that more than six million DALYs are lost and over a quarter of a million deaths occur each year owing to its consumption. There is a need to build on the insights obtained from efforts to reduce cigarette smoking-related harm and to investigate strategies to reduce use of SLT and decrease the substantial associated burden of harm.

## Additional file

Additional file 1:
**Supplementary description of methods and results sections.** (DOCX 281 kb)

## Abbreviations

CI: Confidence intervals
DALYs: Disability-adjusted life years
DHS: Demographic and Health Surveys
GATS: Global Adult Tobacco Survey
ICS: Individual Country Survey
PAF: Population attributable fraction
SEBS: Special Europe Barometer Survey
SLT: Smokeless tobacco
STEPS: STEPwise Approach to Surveillance
TSNA: Tobacco-specific nitrosamines
WHO: World Health Organization
