# Supplementary material for: Global burden of disease due to smokeless tobacco consumption in adults: analysis of data from 113 countries
Source: BMC Med. 2015 Aug 17;13:194. doi: 10.1186/s12916-015-0424-2 (PMC4538761; doi:10.1186/s12916-015-0424-2)
Supplement: Additional file 1: — Supplementary description of methods and results sections. (DOCX 281 kb) [file 12916_2015_424_MOESM1_ESM.docx]

Additional file 1: Supplementary description of methods and results sections

Appendix 1: WHO sub-regional country grouping, by region

| **Africa Region D**: Algeria, Angola, Benin, Burkina Faso, Cameroon, Cape Verde, Chad, Comoros, Equatorial Guinea, Gabon, Gambia, Ghana, Guinea-Bissau, Liberia, Madagascar, Mali, Mauritania, Mauritius, Niger, Nigeria, Sao Tome and Principe, Senegal, Seychelles, Sierra Leone, Togo  **Region E:** Botswana, Burundi, Central African Republic, Congo, Cote d’lvoire, Democratic Republic of Congo, Eritrea, Ethiopia, Kenya, Lesotho, Malawi, Mozambique, Namibia, Rwanda, South Africa, Swaziland, Uganda, Tanzania, Zambia, Zimbabwe  **The Americas Region A:** Canada, USA, Cuba  **Region B:** Antigua and Barbuda, Argentina, Bahamas, Barbados, Belize, Brazil, Chile, Colombia, Costa Rica, Dominica, Dominican Republic, El Salvador, Grenada, Guyana, Honduras, Jamaica, Mexico, Panama, Paraguay, Saint Kitts and Nevis, Saint Lucia, Saint Vincent and the Grenadines, Suriname, Trinidad and Tobago, Uruguay, Venezuela.  **Region D:** Bolivia, Ecuador, Guatemala, Haiti, Nicaragua, Peru  **Eastern Mediterranean Region B:** Bahrain, Iran, Jordan, Kuwait, Lebanon, Libya, Oman, Qatar, Saudi Arabia, Syrian Arab Republic, Tunisia, United Arab Emirates  **Region D:** Afghanistan, Djibouti, Egypt, Iraq, Morocco, Pakistan, Somalia, Sudan, Yemen  **Europe Region A:** Andorra, Austria, Belgium, Croatia, Cyprus, Czech Republic, Denmark, Finland, France, Germany, Greece, Iceland, Ireland, Israel, Italy, Luxembourg, Malta, Monaco, Netherlands, Norway, Portugal, San Marino, Slovenia, Spain, Sweden, Switzerland, UK  **Region B:** Albania, Armenia, Azerbaijan, Bosnia and Herzegovina, Bulgaria, Georgia, Kyrgyzstan, Poland, Romania, Serbia and Montenegro, Slovakia, Tajikistan, Former Yugoslav Republic of Macedonia, Turkey, Turkmenistan, Uzbekistan  **Region C:** Belarus, Estonia, Hungary, Kazakhstan, Latvia, Lithuania, Republic of Moldova, Russian Federation, Ukraine  **Southeast Asia Region: Region B:** Indonesia, Sri Lanka, Thailand  **Region D:** Bangladesh, Bhutan, North Korea, India, Maldives, Myanmar (Burma), Nepal, Timor Leste  **Western Pacific Region: Region A:** Australia, Brunei, Japan, New Zealand, Singapore  **Region B:**Cambodia, China, Cook Islands, Fiji, Kiribati, Laos, Malaysia, Marshall Islands, Micronesia, Mongolia, Nauru, Niue, Palau, Papua New Guinea, Philippines, South Korea, Samoa, Solomon Islands, Tonga, Tuvalu, Vanuatu, Vietnam  Regions are categorized as follows (WHO-approved classifications): A= very low child mortality and very low adult mortality; B=low child mortality and low adult mortality; C=low child mortality and high adult mortality; D= high child mortality and high adult mortality; E=high child mortality and very high adult mortality.  (Adapted from WHO)([1](#_ENREF_1)) |
| --- |

**Appendix 2: Search strategy for the prevalence of smokeless tobacco use**

*Search terminologies:*

Boolean searching techniques were employed using key words; ‘smokeless tobacco’, non-burnt tobacco’, ’spit tobacco’, ‘chewing tobacco’, ‘oral tobacco’,’Gutka’, ‘Naswar’, ‘Nas’, ‘Mishri’, ‘Khiwam’, ‘Zarda’, ‘Mawa’, ‘Pan Masala’, ‘Gudhakhu’, ‘Tuibur’, ‘Shamma’, ’Gul’ and ‘Snuff’ etc. combined with names of countries and regions in different search engines described below. (An example of search terminologies and combinations is given below).

1. smokeless tobacco.mp. or tobacco, smokeless/
2. (oral* adj3 tobacco).mp. [mp=title, abstract, original title, name of substance word, subject heading word, keyword heading word, protocol supplementary concept word, rare disease supplementary concept word, unique identifier]
3. (chew* adj3 tobacco).mp. [mp=title, abstract, original title, name of substance word, subject heading word, keyword heading word, protocol supplementary concept word, rare disease supplementary concept word, unique identifier]
4. (spit* adj3 tobacco).mp. [mp=title, abstract, original title, name of substance word, subject heading word, keyword heading word, protocol supplementary concept word, rare disease supplementary concept word, unique identifier]
5. (dip* adj3 tobacco).mp. [mp=title, abstract, original title, name of substance word, subject heading word, keyword heading word, protocol supplementary concept word, rare disease supplementary concept word, unique identifier]
6. gutk?a.mp.
7. kiwam.mp.
8. zarda.mp.
9. mawa.mp.
10. tuibur.mp.
11. shamma.mp.
12. gul.mp.
13. snuf*.mp.
14. snus.mp.
15. chimo.mp.
16. rape.mp.
17. iqmik.mp.
18. toombak.mp.
19. tumbaku.mp.
20. mishri.mp.
21. m?sheri.mp. [mp=title, abstract, original title, name of substance word, subject heading word, keyword heading word, protocol supplementary concept word, rare disease supplementary concept word, unique identifier]
22. n?swar.mp. [mp=title, abstract, original title, name of substance word, subject heading word, keyword heading word, protocol supplementary concept word, rare disease supplementary concept word, unique identifier]
23. (p?an adj3 (masala or quid)).mp. [mp=title, abstract, original title, name of substance word, subject heading word, keyword heading word, protocol supplementary concept word, rare disease supplementary concept word, unique identifier]
24. gudak?u.mp. [mp=title, abstract, original title, name of substance word, subject heading word, keyword heading word, protocol supplementary concept word, rare disease supplementary concept word, unique identifier]
25. k?aini.mp. [mp=title, abstract, original title, name of substance word, subject heading word, keyword heading word, protocol supplementary concept word, rare disease supplementary concept word, unique identifier]
26. (maras adj3 (powder or tobacco)).mp. [mp=title, abstract, original title, name of substance word, subject heading word, keyword heading word, protocol supplementary concept word, rare disease supplementary concept word, unique identifier]
27. (quid adj3 (betel or tobacco)).mp. [mp=title, abstract, original title, name of substance word, subject heading word, keyword heading word, protocol supplementary concept word, rare disease supplementary concept word, unique identifier]
28. ((twist or plug) adj3 tobacco).mp. [mp=title, abstract, original title, name of substance word, subject heading word, keyword heading word, protocol supplementary concept word, rare disease supplementary concept word, unique identifier]
29. ((loose leaf or tablet* or toothpaste) adj3 tobacco).mp. [mp=title, abstract, original title, name of substance word, subject heading word, keyword heading word, protocol supplementary concept word, rare disease supplementary concept word, unique identifier]
30. ((pouch* or mix* or powder*) adj3 tobacco).mp. [mp=title, abstract, original title, name of substance word, subject heading word, keyword heading word, protocol supplementary concept word, rare disease supplementary concept word, unique identifier]
31. OR/1-30
32. Epidemiolog$
33. Prevalence$
34. Frequency
35. Survey
36. OR/32-35
37. Region (x) OR Country (x)
38. 31 AND 36 AND 37

*Databases:*

Search engines included Medline, Embase, Web of Science, CINAHIL, Pakmed, Informit, Ingenta Connect, Global Health, AJLOL African Journals Online, Airiti Inc, Academic Search, Pubget, OALster, IndMED, LILACS and Cochrane Database were used. Moreover, Google Scholar, Pubmed Database (January 1946 - February 2015) and key websites such as (World Health Organisation (WHO), Action on Smoking and Health (ASH) UK, Action on Smoking and Health (ASH) USA, National Institutes of Health (NIH), and Centres for Disease Control and Prevention (CDC) were also searched.

*Language:*

No restrictions

*Inclusion criteria:*

Prevalence of smokeless tobacco was extracted from only those surveys and studies, which were designed to investigate the prevalence of smokeless tobacco

**Appendix 3: Search strategy for literature review of disease outcomes associated with smokeless tobacco use**

*Databases*

MEDLINE, EMBASE, PsycINFO, CINAHL Plus, Web of Science (including Conference Proceedings Citation Index, accessed via Web of Science™ Core Collection), Scopus, Cochrane Library, African Journals Online (AJOL), Latin American and Caribbean Health Sciences Literature (LILACS), WHO Index Medicus of the Eastern Mediterranean Region (IMEMR), WHO Index Medicus of the South-East Asian Region (IMSEAR), PakMediNet, IndMED, ProQuest Dissertations & Theses A&I, EThOS, Open Grey. Reference lists of selected articles were also used to identify relevant studies.

*Main Keywords and Phrases*

The search for this review was conducted by combining an exhaustive list of terms for smokeless tobacco with terms for specific cancers and cardiovascular disease outcomes. The search strategy used for each disease outcome is provided below.

1. Smokeless tobacco and cancer (*MEDLINE via OVID search strategy*) - for the period 1946 to July Week 2, 2014
2. smokeless tobacco.mp. or tobacco, smokeless/
3. (oral* adj3 tobacco).mp. [mp=title, abstract, original title, name of substance word, subject heading word, keyword heading word, protocol supplementary concept word, rare disease supplementary concept word, unique identifier]
4. (chew* adj3 tobacco).mp. [mp=title, abstract, original title, name of substance word, subject heading word, keyword heading word, protocol supplementary concept word, rare disease supplementary concept word, unique identifier]
5. (spit* adj3 tobacco).mp. [mp=title, abstract, original title, name of substance word, subject heading word, keyword heading word, protocol supplementary concept word, rare disease supplementary concept word, unique identifier]
6. (dip* adj3 tobacco).mp. [mp=title, abstract, original title, name of substance word, subject heading word, keyword heading word, protocol supplementary concept word, rare disease supplementary concept word, unique identifier]
7. gutk?a.mp.
8. kiwam.mp.
9. zarda.mp.
10. mawa.mp.
11. tuibur.mp.
12. shamma.mp.
13. gul.mp.
14. snuf*.mp.
15. snus.mp.
16. chimo.mp.
17. rape.mp.
18. iqmik.mp.
19. toombak.mp.
20. tumbaku.mp.
21. mishri.mp.
22. m?sheri.mp. [mp=title, abstract, original title, name of substance word, subject heading word, keyword heading word, protocol supplementary concept word, rare disease supplementary concept word, unique identifier]
23. n?swar.mp. [mp=title, abstract, original title, name of substance word, subject heading word, keyword heading word, protocol supplementary concept word, rare disease supplementary concept word, unique identifier]
24. (p?an adj3 (masala or quid)).mp. [mp=title, abstract, original title, name of substance word, subject heading word, keyword heading word, protocol supplementary concept word, rare disease supplementary concept word, unique identifier]
25. gudak?u.mp. [mp=title, abstract, original title, name of substance word, subject heading word, keyword heading word, protocol supplementary concept word, rare disease supplementary concept word, unique identifier]
26. k?aini.mp. [mp=title, abstract, original title, name of substance word, subject heading word, keyword heading word, protocol supplementary concept word, rare disease supplementary concept word, unique identifier]
27. (maras adj3 (powder or tobacco)).mp. [mp=title, abstract, original title, name of substance word, subject heading word, keyword heading word, protocol supplementary concept word, rare disease supplementary concept word, unique identifier]
28. (quid adj3 (betel or tobacco)).mp. [mp=title, abstract, original title, name of substance word, subject heading word, keyword heading word, protocol supplementary concept word, rare disease supplementary concept word, unique identifier]
29. ((twist or plug) adj3 tobacco).mp. [mp=title, abstract, original title, name of substance word, subject heading word, keyword heading word, protocol supplementary concept word, rare disease supplementary concept word, unique identifier]
30. ((loose leaf or tablet* or toothpaste) adj3 tobacco).mp. [mp=title, abstract, original title, name of substance word, subject heading word, keyword heading word, protocol supplementary concept word, rare disease supplementary concept word, unique identifier]
31. ((pouch* or mix* or powder*) adj3 tobacco).mp. [mp=title, abstract, original title, name of substance word, subject heading word, keyword heading word, protocol supplementary concept word, rare disease supplementary concept word, unique identifier]
32. 1 or 2 or 3 or 4 or 5 or 6 or 7 or 8 or 9 or 10 or 11 or 12 or 13 or 14 or 15 or 16 or 17 or 18 or 19 or 20 or 21 or 22 or 23 or 24 or 25 or 26 or 27 or 28 or 29
33. mouth.mp.
34. ((oral$ or bucca$ or 'oral cavit$' or (oral adj mucosa$) or (mouth adj mucosa$) or lip or lips or tongue$ or gingiv$ or palat$ or cheek$ or 'intra oral$' or intraoral$ or gum or labial$) adj3 (tumor$ or tumour$ or cancer$ or carcinoma$ or carcinogen$ or neoplas$ or malignan$ or metastas$ or dysplas$ or lesion$ or ulcer$)).mp.
35. Exp Pharynx/di
36. Pharynx$.ab,ti.
37. Pharyn$ adj5 (tumor$ or tumour$ or cancer$ or carcinoma$ or carcinogen$ or neoplas$ or malignan$ or metasta$ or dysplas$ or lesion$ or ulcer$).tw,ot.
38. ((esophag$ or oesophag$) adj5 (tumor$ or cancer$ or carcinoma$ or carcinogen$ or neoplas$ or malignan$ or metasta$ or dysplas$ or lesion$ or ulcer$)).tw,ot.
39. ((pancrea$ or pancreas$) adj5 (tumor$ or cancer$ or carcinoma$ or carcinogen$ or neoplas$ or malignan$ or metasta$ or dysplas$ or lesion$ or ulcer$)).tw,ot.
40. (lung$ adj5 (tumor$ or cancer$ or carcinoma$ or carcinogen$ or neoplas$ or malignan$ or metasta$ or dysplas$ or lesion$ or ulcer$)).tw,ot.
41. Smokeless tobacco and cardiovascular diseases (*MEDLINE via OVID search strategy*) - for the period 1946 to July Week 2, 2014

1. smokeless tobacco*.mp. or Tobacco, Smokeless/

2. (oral* adj3 tobacco*).mp. [mp=title, abstract, original title, name of substance word, subject heading word, keyword heading word, protocol supplementary concept word, rare disease supplementary concept word, unique identifier]

3. (chew* adj3 tobacco*).mp. [mp=title, abstract, original title, name of substance word, subject heading word, keyword heading word, protocol supplementary concept word, rare disease supplementary concept word, unique identifier]

4. (spit* adj3 tobacco*).mp. [mp=title, abstract, original title, name of substance word, subject heading word, keyword heading word, protocol supplementary concept word, rare disease supplementary concept word, unique identifier]

5. (dip* adj3 tobacco*).mp. [mp=title, abstract, original title, name of substance word, subject heading word, keyword heading word, protocol supplementary concept word, rare disease supplementary concept word, unique identifier]

6. gutk?a.mp.

7. kiwam.mp.

8. zarda.mp.

9. mawa.mp.

10. tuibur.mp.

11. shamma.mp.

12. gul.mp.

13. snuf*.mp.

14. snus.mp.

15. chimo.mp.

16. rapê.mp.

17. iqmik.mp.

18. toombak.mp.

19. tumbaku.mp.

20. mishri.mp.

21. m?sheri.mp. [mp=title, abstract, original title, name of substance word, subject heading word, keyword heading word, protocol supplementary concept word, rare disease supplementary concept word, unique identifier]

22. n?swar.mp. [mp=title, abstract, original title, name of substance word, subject heading word, keyword heading word, protocol supplementary concept word, rare disease supplementary concept word, unique identifier]

23. (p?an adj3 (masala or quid)).mp. [mp=title, abstract, original title, name of substance word, subject heading word, keyword heading word, protocol supplementary concept word, rare disease supplementary concept word, unique identifier]

24. gudak?u.mp. [mp=title, abstract, original title, name of substance word, subject heading word, keyword heading word, protocol supplementary concept word, rare disease supplementary concept word, unique identifier]

25. k?aini.mp. [mp=title, abstract, original title, name of substance word, subject heading word, keyword heading word, protocol supplementary concept word, rare disease supplementary concept word, unique identifier]

26. (maras adj3 (powder or tobacco*)).mp. [mp=title, abstract, original title, name of substance word, subject heading word, keyword heading word, protocol supplementary concept word, rare disease supplementary concept word, unique identifier]

27. (quid adj3 (betel or tobacco*)).mp. [mp=title, abstract, original title, name of substance word, subject heading word, keyword heading word, protocol supplementary concept word, rare disease supplementary concept word, unique identifier]

28. ((twist* or plug*) adj3 tobacco*).mp. [mp=title, abstract, original title, name of substance word, subject heading word, keyword heading word, protocol supplementary concept word, rare disease supplementary concept word, unique identifier]

29. ((loose leaf or toothpaste*) adj3 tobacco*).mp. [mp=title, abstract, original title, name of substance word, subject heading word, keyword heading word, protocol supplementary concept word, rare disease supplementary concept word, unique identifier]

30. ((pouch* or mix* or powder*) adj3 tobacco*).mp. [mp=title, abstract, original title, name of substance word, subject heading word, keyword heading word, protocol supplementary concept word, rare disease supplementary concept word, unique identifier]

31. 1 or 2 or 3 or 4 or 5 or 6 or 7 or 8 or 9 or 10 or 11 or 12 or 13 or 14 or 15 or 16 or 17 or 18 or 19 or 20 or 21 or 22 or 23 or 24 or 25 or 26 or 27 or 28 or 29

32. exp Cardiovascular Diseases/ or cardiovascular disease*.mp.

33. exp Myocardial Infarction/ or myocardial infarc*.mp.

34. heart attack*.mp.

35. exp Heart Arrest/ or heart arrest*.mp.

36. exp Coronary Disease/ or exp Coronary Artery Disease/ or coronary disease*.mp.

37. coronary event*.mp.

38. cardio?vascular mortalit*.mp.

39. cardiac mortalit*.mp.

40. cardio?vascular death*.mp.

41. exp Death, Sudden, Cardiac/ or cardiac death*.mp.

42. exp Cerebrovascular Disorders/ or cerebrovascular disorder*.mp.

43. cerebro?vascular accident*.mp.

44. cerebro?vascular event*.mp.

45. cerebro?vascular disease*.mp.

46. exp Stroke/ or stroke*.mp.

47. brain isch?emia.mp. or exp Brain Ischemia/

48. exp Intracranial Hemorrhages/ or exp Cerebral Hemorrhage/ or intracranial h?emorrhag*.mp.

49. 31 or 32 or 33 or 34 or 35 or 36 or 37 or 38 or 39 or 40 or 41 or 42 or 43 or 44 or 45 or 46 or 47

50. 30 and 48

*Language*

No restrictions on language

*Study selection criteria*

- Participants/population - Not restricted by age, gender or country
- Types of study - Cohort studies and case-control studies that reported cancers and cardiovascular disease outcomes in smokeless tobacco users were included. Cross*-*sectional studies, case series and case reports were excluded.
- Exposure - Any use of smokeless tobacco (current and past); If an identified study included users of both smoked and smokeless forms of tobacco, then the presented risk estimate should have been adjusted for smoking.
- Outcome:
  - Cancers - Studies reporting risk estimates for cancers of oral cavity or oropharynx, hypopharynx, nasopharynx, larynx and lip, oesophagus, lungs and pancreas among smokeless tobacco users were included.
  - Cardiovascular diseases *-* Studies reporting risk estimates for ischaemic heart disease (IHD), and stroke among smokeless tobacco users were included. Studies that only reported ‘intermediate’ cardiovascular outcomes such as blood pressure or lipid levels were excluded from the review.

Appendix 4: Risk of diseases applied to different WHO regions and the justification

| **Region** | Risk of mouth, pharyngeal and oesophageal cancers and ischaemic heart disease | |
| --- | --- | --- |
|  | **Risk estimates (RR/OR applied)** | **Justification** |
| **Africa D** | For cancers, non-specific global estimates (oral 3.43; pharyngeal 2.2; oesophageal 2.17) and for ischaemic heart disease, INTERHEART study estimates (1.57) | No country specific risk data available from the region but there is reported use of Tambook (Chad) and snuff (Nigeria, Algeria, Ghana); both products have high pH and TSNA. |
| **Africa E** | For cancers, non-specific global estimates (oral 3.43; pharyngeal 2.2; oesophageal 2.17) and for ischaemic heart disease, INTERHEART study estimates (1.57) | No country specific risk data available from the region but there is reported use of snuff (South Africa, Uganda and Tanzania) products with high pH and TSNA. Besides, Guthka is also used in East Africa (Uganda, Tanzania) among those of South Asian-origin. |
| **Americas A** | For cancers, country-specific (US) estimates (all cancers 1.0) and for ischaemic heart disease, INTERHEART study estimates (1.57) | Some risk data available from US-based studies. Same SLT products are used both in the US and Canada. |
| **Americas B** | For cancers, country-specific (US) estimates for Mexico (all cancers 1.0) and non-specific global estimates (oral 3.43; pharyngeal 2.2; oesophageal 2.17) for the rest. For ischaemic heart disease, INTERHEART study estimates (1.57) | No country specific risk data available. SLT used in Mexico is similar to the US. Chimó consumption has been reported (Venezuela, Colombia) which has a moderate level of pH and TSNA. Brazil reported use of a SLT product called Rapé but little information is available on this product. |
| **Americas D** | No estimates | No country specific risk data available. Little information on the products available. |
| **Eastern Mediterranean B** | For cancers, non-specific global estimates (oral 3.43; pharyngeal 2.2; oesophageal 2.17) and for ischaemic heart disease, INTERHEART study estimates (1.57) | No country specific risk data available. A commonly used SLT product used in this region is Shammah, which (due to its preparation) is likely to be similar to Tambook in its toxic properties. |
| **Eastern Mediterranean D** | For cancers, country-specific (India) estimates (oral 5.12; pharyngeal 2.6; oesophageal 2.57) for Pakistan and non-specific global estimates for the rest (oral 3.43; pharyngeal 2.2; oesophageal 2.17). For ischaemic heart disease, INTERHEART study estimates (1.57) | No good country specific risk data available. SLT products used in Pakistan include Nass, Zarda and Guthka, associated with high PH and TSNA. Tambook is used in Sudan also with high pH and TSNA. Shammah is used in Yemen. |
| **Europe A** | For cancers, country-specific (Sweden) estimates (oral & pharyngeal 1.0; oesophageal 1.23) for Europe A except for the UK where country-specific (India) estimates (oral 5.12; pharyngeal 2.6; oesophageal 2.57) are applied. For ischaemic heart disease, country-specific (Sweden) estimates (1.0) for Europe A except for the UK where INTERHEART study estimates (1.57) were applied | Considerable country specific risk data available on snus products used in Nordic countries. Little information on the type of SLT products and the associated risks from the rest of Europe expect the UK where Zarda, Guthka, and Khaini are used by those of South Asian-origin**.** |
| **Europe B** | For cancers, non-specific global estimates (oral 3.43; pharyngeal 2.2; oesophageal 2.17) and for ischaemic heart disease, INTERHEART study estimates (1.57) | No country specific risk data available. However, there is reported and widespread use of Nass in central Asian countries including Uzbekistan, Kyrgyzstan, and Tajikistan – an SLT product with very high pH and TSNA levels. |
| **Europe C** | No estimates applied | No country specific risk data available. Little information on the products available. |
| **South-East Asia B** | For cancers, country-specific (India) estimates (oral 5.12; pharyngeal 2.6; oesophageal 2.57) and for ischaemic heart disease, INTERHEART study estimates (1.57) | No country specific risk data available. However, a wide range of SLT products including betel quid use has been reported in the region (Indonesia, Thailand, Sri Lanka) which are similar to those used in India and Bangladesh. |
| **South-East Asia D** | For cancers, country-specific (India) estimates (oral 5.12; pharyngeal 2.6; oesophageal 2.57) and for ischaemic heart disease, INTERHEART study estimates (1.57) | Considerable country-specific risk data available mainly from India where common SLT products include Khaini, Zarda, and Guthka. Some data also available from Pakistan and Bangladesh where Nass, Guthka, Zarda and betel quid with tobacco are some of the common products. |
| **Western Pacific A** | For cancers, no estimates applied  For ischaemic heart disease, INTERHEART study estimates (1.57) | No country specific risk data available. Little information on the products available. Included in the INTERHEART study |
| **Western Pacific B** | For cancers, country specific (India) estimates (oral 5.12; pharyngeal 2.6; oesophageal 2.57) applied except for China and Mongolia where no estimates were applied  For ischaemic heart disease, INTERHEART study estimates (1.57) | No country specific risk data available. However reported use of the SLT products (betel quid with tobacco and areca nut) in the region (Laos, Cambodia, Malaysia, Vietnam), which are similar to those, used in South East Asia. There is no information on the SLT products used in Mongolia and China. |

Appendix 5: Flow diagrams (PRISMA) of the selection process of articles included in the SLT prevalence and the two reviews to assess risk

5a: Smokeless tobacco prevalence

**Only one prevalence report was included for one country. Latest national prevalence data collected as part of an international or regional survey was preferred over an older isolated national or a sub-national survey*

Studies included in quantitative synthesis*
(n =55)

Studies included in qualitative synthesis
(n = 55)

Full-text articles assessed for eligibility
(n = 99)

Records excluded
(n = 926)

Records screened
(n = 1025)

Records after duplicates removed
(n = 1025)

Additional records identified through other sources
(n = 54)

## Identification

## Eligibility

## Included

## Screening

Records identified through database searching
(n = 976)

Full-text articles excluded, with reasons
(n = 44)

National but old surveys (n = 7)

Population sub-group surveys (n = 7)

Findings reported elsewhere (n =15)

Subnational surveys (n = 15)

Studies included in quantitative synthesis (meta-analysis)
(n = 39)

Studies included in qualitative synthesis
(n = 123)

Full-text articles assessed for eligibility
(n = 166)

Records excluded
(n = 6061)

Records screened
(n = 6227)

Records after duplicates removed
(n = 6227)

Additional records identified through other sources
(n = 17)

## Identification

## Eligibility

## Included

## Screening

Records identified through database searching
(n = 6661)

5b: Cancers

Full-text articles excluded, with reasons
(n = 84)

Not exclusive SLT use/did not report adjusted Ors/RRs (n = 43)

Did not meet exposure criteria (n = 21)

Combined outcome reporting (n = 15)

Gastric cancer (n = 1)

Bladder cancer (n = 1)

Only cancer deaths reported as outcomes

(n = 3)

5c: Cardiovascular diseases

**Included studies have reported multiple CVD outcomes*

Studies included in quantitative synthesis (meta-analysis)*
(n = 14)

Studies included in qualitative synthesis
(n = 14)

Full-text articles assessed for eligibility
(n = 24)

Records excluded
(n = 2364)

Records screened
(n = 2386)

Records after duplicates removed
(n = 2386)

Additional records identified through other sources
(n = 0)

## Identification

## Eligibility

## Included

## Screening

Records identified through database searching
(n = 2407)

Full-text articles excluded, with reasons
(n = 10)

Pooled analysis of 8 cohorts (n = 2)

Not exclusive SLT use (n = 1)

Combined outcome reporting (n = 1)

Only CVD deaths reported as outcomes (n = 6)

Appendix 6: Random effects models (excluded) showing relative risks for laryngeal, lung and pancreatic cancers and stroke for smokeless tobacco use

6a. Laryngeal cancer

**
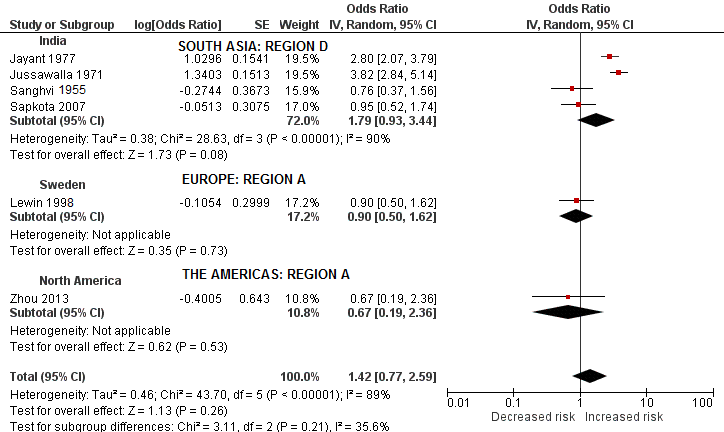
**

6b. Lung cancer

**
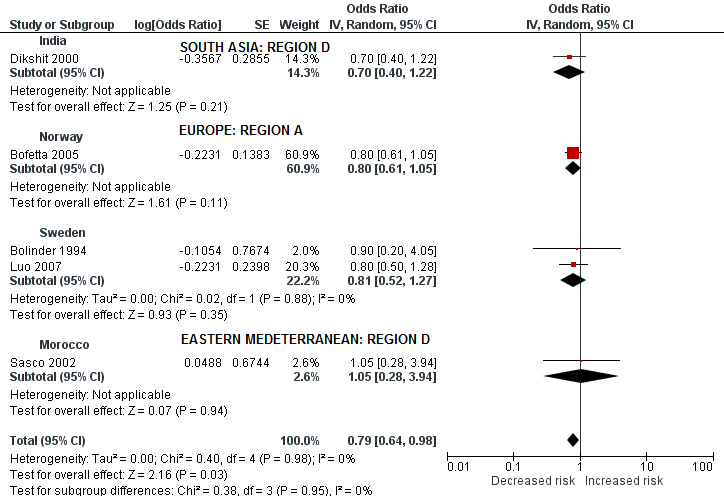
**

6c. Pancreatic cancer

**
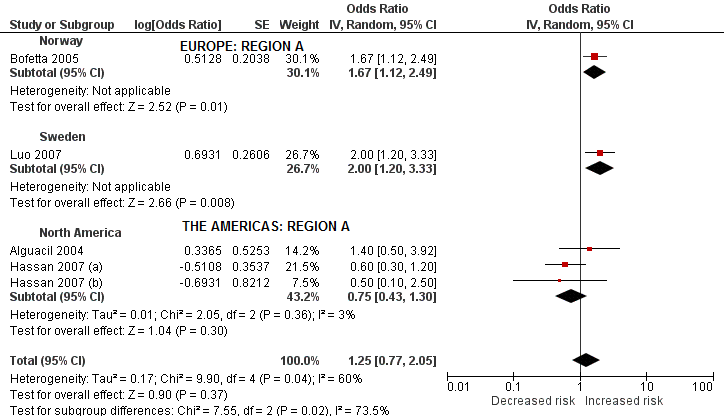
**

*Hassan 2007 (a): Chewing tobacco users*

*Hassan 2007 (b): Snuff users*

6d. Stroke

**
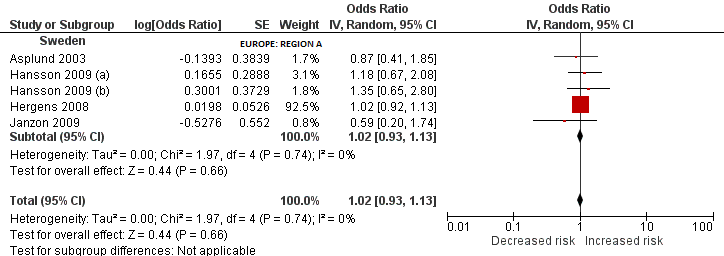
**

*Hansson 2009 (a): Current users*

*Hansson 2009 (b): Former users*

**References of appendix:**

1. Mutangadura GB. World Health Report 2002: Reducing Risks, Promoting Healthy Life: World Health Organization, Geneva, 2002, 250 pages, US $13.50, ISBN 9-2415-6207-2. No longer published by Elsevier; 2004.
